# MEAC: A Multi-Scale Edge-Aware Convolution Module for Robust Infrared Small-Target Detection

**DOI:** 10.3390/s25144442

**Published:** 2025-07-16

**Authors:** Jinlong Hu, Tian Zhang, Ming Zhao

**Affiliations:** 1 Institute of Seismology, China Earthquake Administration, Wuhan 430071, China; hujinlong23@mails.ucas.ac.cn; 2 Northwest Land and Resource Research Center, Shaanxi Normal University, Xi’an 710119, China; 2019147@snnu.edu.cn; 3 School of Cybersecurity and Informatization, Wuxi University, Wuxi 214105, China

**Keywords:** infrared small-target detection, Multi-Scale Edge-Aware Convolution (MEAC), multi-scale dilated convolution, differential Gaussian edge extraction, feature fusion, attention mechanisms

## Abstract

Infrared small-target detection remains a critical challenge in military reconnaissance, environmental monitoring, forest-fire prevention, and search-and-rescue operations, owing to the targets’ extremely small size, sparse texture, low signal-to-noise ratio, and complex background interference. Traditional convolutional neural networks (CNNs) struggle to detect such weak, low-contrast objects due to their limited receptive fields and insufficient feature extraction capabilities. To overcome these limitations, we propose a Multi-Scale Edge-Aware Convolution (MEAC) module that enhances feature representation for small infrared targets without increasing parameter count or computational cost. Specifically, MEAC fuses (1) original local features, (2) multi-scale context captured via dilated convolutions, and (3) high-contrast edge cues derived from differential Gaussian filters. After fusing these branches, channel and spatial attention mechanisms are applied to adaptively emphasize critical regions, further improving feature discrimination. The MEAC module is fully compatible with standard convolutional layers and can be seamlessly embedded into various network architectures. Extensive experiments on three public infrared small-target datasets (SIRSTD-UAVB, IRSTDv1, and IRSTD-1K) demonstrate that networks augmented with MEAC significantly outperform baseline models using standard convolutions. When compared to eleven mainstream convolution modules (ACmix, AKConv, DRConv, DSConv, LSKConv, MixConv, PConv, ODConv, GConv, and Involution), our method consistently achieves the highest detection accuracy and robustness. Experiments conducted across multiple versions, including YOLOv10, YOLOv11, and YOLOv12, as well as various network levels, demonstrate that the MEAC module achieves stable improvements in performance metrics while slightly increasing computational and parameter complexity. These results validate the MEAC module’s significant advantages in enhancing the detection of small and weak objects and suppressing interference from complex backgrounds. These results validate MEAC’s effectiveness in enhancing weak small-target detection and suppressing complex background noise, highlighting its strong generalization ability and practical application potential.

## 1. Introduction

**Figure 1:**
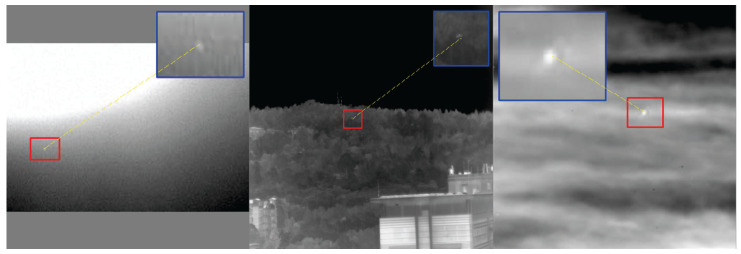
Infrared target image.

Infrared target detection technology detects targets based on differences in the infrared radiation emitted by the targets and their surroundings [1]. Infrared small-target detection (IRST) is a valuable and highly challenging research field with significant applications in military reconnaissance, environmental monitoring, forest-fire prevention, search and rescue, and other areas [2,3,4,5,6,7]. However, IRST faces a series of unique challenges that differ from those of general target detection. Small infrared targets are typically extremely small, often occupying only a few pixels in an image. They lack texture and shape information and often appear as blurry spots. Images have low signal-to-noise ratios and are susceptible to atmospheric and sensor noise. Complex backgrounds, such as clouds, ground clutter, and building edges, are similar in appearance to small targets and easily cause false alarms (Figure 1). These factors make accurately and robustly detecting small targets from complex infrared images difficult.

Traditional signal processing-based IRST methods primarily rely on local high-contrast or sparse features exhibited by small targets in infrared images. Typical techniques include local contrast measures (LCM [8]) and its improvements (e.g., NLCM [9]), morphological filtering (e.g., Top-Hat transformation [6]), and image decomposition methods based on low-rank sparse decomposition (e.g., IPI [10], NIPPS [11]). While these methods offer computational efficiency, they lack robustness in complex, dynamic environments and fail to generalize across diverse scenarios.

In recent years, deep learning, especially convolutional neural networks (CNNs), has made significant progress in computer vision. Its powerful end-to-end feature learning capabilities have been widely applied to IRST tasks. However, standard convolution operations have fixed receptive fields and shared weights, which makes it difficult to fully extract the features of small, textureless, and weak targets. Additionally, while common pooling operations are helpful for obtaining high-level semantic information, they inevitably lose spatial details, which is unacceptable for small targets that rely on precise localization.

To improve CNN performance in small-object detection, researchers have proposed the following strategies: First, introduce multi-scale convolutions (e.g., parallel hollow convolutions or pyramid structures) to expand the receptive field [12,13,14]. Second, use multi-scale hierarchical connections within the same network block to obtain richer hierarchical features [15]. Third, leverage spatial adaptive or dynamic convolutions (e.g., deformable convolutions [16], Involution [17], CondConv [18], Dynamic Conv [19], etc.) to improve adaptability to target deformations and complex backgrounds. A fourth strategy is to combine feature fusion and attention mechanisms (e.g., feature pyramids, channel/spatial attention) to adaptively emphasize key information for small targets and suppress background noise [20,21]. While the above research has produced positive results in general small-target scenarios, these solutions have limitations when dealing with infrared small targets. On the one hand, although hollow convolutions or multi-scale methods can enhance the receptive field of context, they lack the ability to specifically extract the high-contrast edge features of infrared small targets. Second, deformable or dynamic convolution focuses on dynamically adjusting spatial sampling positions or global features but does not fuse two types of key information—“multi-scale context + edge contrast”—at the basic convolution unit level. Therefore, existing methods struggle to integrate these complementary features while preserving spatial details, thereby limiting the network’s ability to recognize small infrared targets.

To address these challenges, this paper proposes a novel multi-scale, edge-aware convolution, or MEAC, module. The MEAC module uses a parallel processing and feature fusion structure to create a convolution unit that is better suited for extracting small target features in the infrared spectrum. The module combines three parallel feature streams: the identity branch retains the original local features; the multi-scale spatial convolution branch aggregates context information from different scales while maintaining spatial resolution; and the differential Gaussian edge branch captures high-contrast boundaries between targets and backgrounds. The differential Gaussian edge extraction branch directly captures high-contrast boundaries between targets and backgrounds. After fusing these complementary features, the MEAC module introduces channel and spatial attention to adaptively enhance key feature channels.

Designed as a universal building block, the MEAC module can easily replace the standard convolution layer in existing CNN architectures, enhancing their perception and processing capabilities for small infrared targets without altering the network’s overall structure. It is worth noting that the MEAC design fully considers the minimum increase in parameters and computation. This enables MEAC to be easily deployed on various IoT platforms while maintaining high performance. This makes MEAC significant for applications in edge computing and embedded devices [22].

To verify the effectiveness of the proposed MEAC module, we conducted extensive experiments on multiple public infrared small-target datasets and multiple mainstream object detection models. The results demonstrate that networks incorporating the MEAC module achieve significantly superior detection performance compared to networks using standard convolutions. These networks effectively improve detection accuracy and robustness for weak, small targets in complex backgrounds.

## 2. Related Work

This section first reviews the development of infrared search and tracking (IRST) methods in the era of deep learning. Then, it analyzes the applicability and shortcomings of various convolutional improvement techniques in small-object detection, particularly in infrared scenarios involving small objects.

### 2.1. Deep Learning-Based Infrared Small-Object Detection Methods

As deep learning has rapidly developed, researchers have begun exploring the application of general-purpose object detection or semantic segmentation frameworks in the IRST domain. They have adapted and improved these frameworks to suit the characteristics of infrared small targets. Overall, the relevant work can be categorized into the following types:

1. Adaptation and improvement of general-purpose detection/segmentation frameworks
  Based on classic object detection networks such as Faster R-CNN [23,24,25] and the YOLO series [26,27], Hao et al. utilized super-resolution technology for infrared image preprocessing to enhance weak target features, and then combined it with the YOLO object detection model for detection [28]. Zhang proposed a feature fusion-based infrared weak target detection method based on Faster R-CNN, which improves the performance of infrared weak target detection by integrating feature information from different scales [29].
2. Feature Fusion Strategy
  Small targets are prone to missing information after undergoing multiple convolutions and pooling in deep networks. To address this issue, researchers have proposed multi-layer feature fusion modules that combine high-resolution detail information from the bottom layer with semantic features from the deeper layers. Tong et al. [30] introduced an enhanced asymmetric attention (EAA) module that substantially improves the feature representation of small infrared targets through same-layer feature exchanges and cross-layer feature fusion. Additionally, methods such as Experiment [31], DFN [32], and SENet [33] achieve learnable fusion at different levels. However, pure feature fusion often fails to fully recover weak signals of small targets on deep semantic maps and must be combined with targeted feature enhancement strategies.
3. Introduction of Attention Mechanisms
  These mechanisms guide the network to focus on the most critical regions or channels in an image, thereby improving the detection of small objects. Chen et al. proposed the Local Patch Network (LPNet) [34], which integrates global and local attention within the network. Zhang et al. proposed the Infrared Shape Network (ISNet) [35], which includes a bidirectional attention aggregation (TOAA) block to enhance sensitivity to target shape edges. As Transformers have become popular in computer vision, Liu et al. [36] were the first to apply self-attention mechanisms to infrared small-object segmentation. Additionally, Wang proposed an internal attention-aware network (IAANet) [37] with a coarse-to-fine structure to improve the network’s response to weak small targets. While the aforementioned methods have partially addressed the issue of weak infrared small-target signals, most still rely on attention guidance at higher feature levels and lack specialized optimization for basic feature extraction units in convolutional layers.

In summary, deep learning methods have made progress in IRST scenarios; however, most research focuses on feature fusion or attention guidance at the network structure level. The basic feature extraction modules at the convolutional layer have not been specifically designed, resulting in limitations when handling small targets with no texture and weak local signals.

### 2.2. Convolution Improvement Techniques and Their Applicability to the Detection of Small Targets in the Infrared Spectrum

To improve the ability of convolutional neural networks to detect weak targets in complex scenes, the academic community has proposed strategies to enhance the performance of convolutional operators. The following analysis focuses on typical techniques and examines their respective advantages and limitations in infrared small-target detection tasks.

1. Spatially adaptive convolution
  **Deformable Convolutional Networks** DCNs [16]: By learning trainable sampling offsets, the convolution kernel can dynamically align feature locations according to the target’s geometric shape. However, infrared small targets often appear as near-circular or blurry spots with limited geometric deformation potential; thus, the geometric alignment advantage of deformable convolutions has not been fully exploited.
  **Involution** [17]: This method generates independent, learnable convolution kernels for each spatial location, achieving spatially specific filtering and enhancing local flexibility. However, it has not been specifically designed for high-contrast edges or the multi-scale contexts of small infrared targets. It is also not precise enough in suppressing background noise and extracting weak signals.
2. Dynamic and Conditional Convolution
  Conditional convolutions (**CondConv [18], Dynamic Conv [19], and ODConv [38]**) achieve adaptive responses to different inputs by dynamically adjusting the convolution kernel or its weights. Such algorithms can improve the network’s robustness to diverse backgrounds. However, their dynamic mechanisms focus more on adapting overall or large-scale features. They fail to enhance local weak target signals and fully capture the high-contrast edge information of small infrared targets.
3. Feature Enhancement and Fusion Convolution
  **ACmix** [39]: This method integrates convolution operations with self-attention mechanisms within the same module to jointly model global and local features. This enhances the network’s overall feature expression capabilities; however, the module does not include a custom design for edge features or contrast information of small infrared targets. This results in insufficient sensitivity to weak targets.
  **Selective Kernel (SK) Convolution** [40]: This approach uses multi-branch parallel processing to extract features at different scales. This allows the network to dynamically select the most appropriate receptive field based on the input. However, SK convolution only optimizes the multi-scale aspect of traditional convolution and does not process feature textures.
  **Group convolution** [41] is widely used due to its low computational cost. Compared to ordinary convolution, the number of parameters and computational complexity are both reduced by a factor of G, where G is the group size. A special case of group convolution is **depth separable convolution** [42], where the number of groups equals the number of channels. Although group convolutions are efficient, they have limited modeling capabilities for local details and multi-scale context, making them unsuitable for edge extraction and semantic segmentation of small infrared targets.

In summary, different convolution improvement techniques offer various advantages for general object detection and semantic segmentation tasks. However, in scenarios involving the detection of small objects in infrared, it is impossible to meet the following three requirements simultaneously at the convolution layer:

- Local contrast edge features:
  Precise extraction of high-contrast edges inherent to infrared small targets.
- Multi-scale contextual information:
  Acquisition of rich contextual features without sacrificing spatial resolution to distinguish targets from the background.
- Preservation of original local features:
  Ensures that fine-grained spatial details are not overly smoothed or weakened during multiple convolutions and fusions.

## 3. Method

### 3.1. Overall Structure Design

To overcome the limitations of traditional convolutions in multi-scale feature extraction and edge detail modeling, this paper introduces a new convolutional module called Multi-scale Edge-Aware Convolution (MEAC). The MEAC module integrates multi-scale dilated convolution, difference Gaussian edge extraction (DoG edge extraction), and channel–spatial attention mechanisms. These mechanisms enhance the module’s feature expression capabilities, particularly in image tasks involving complex textures or blurred object edges. This improves the module’s robustness. The overall process is divided into four stages:

- Initial channel mapping.
- Multi-dimensional feature extraction.
- Feature concatenation and fusion.
- Output mapping and downsampling.

**Figure 2:**
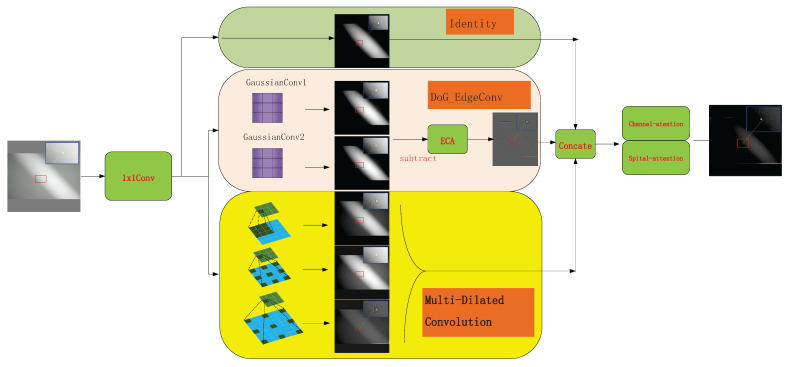
Structure chart.

Figure 2 shows the structural diagram. This module can serve as an alternative to general convolutional modules. The following sections will introduce the design details of each submodule and the overall structural fusion strategy.

### 3.2. Channel Mapping and Multi-Dimensional Feature Extraction

#### 3.2.1. Channel Mapping

Use 1×1 convolution to map *X* from $C_{\mathrm{in}}$ channels to $C_{m}$ (Equation (1)):

(1) $$F=\mathrm{SiLU}\left(\mathrm{BN}(W_{\mathrm{init}}*X)\right),\;W_{\mathrm{init}}\in R^{C_{m}\times C_{\mathrm{in}}\times 1\times 1}$$

where $C_{m}=r\cdot C_{\mathrm{out}}$ (where *r* is the expansion coefficient).

#### 3.2.2. Multi-Dimensional Feature Extraction

Feed $F\in R^{B\times C_{m}\times H\times W}$ into three parallel branches to extract the original local features, the multi-scale cavity context features, and the high-contrast edge features, respectively.

**Figure 3:**
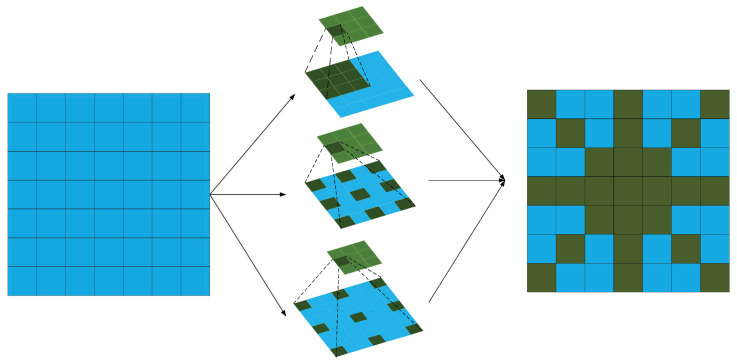
Receptive fields.

**Figure 4:**
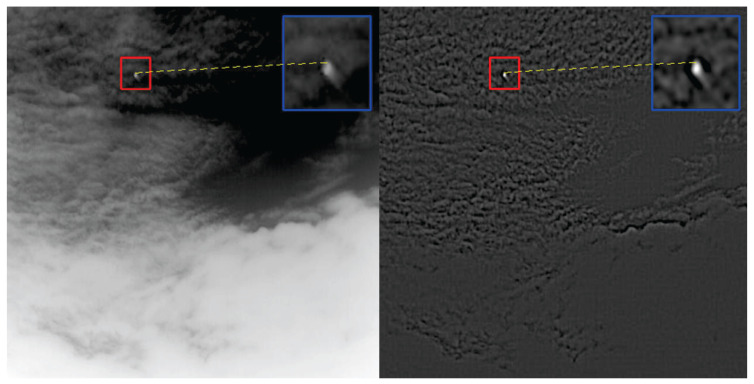
DoG edge extraction image.

1. The feature retention branch (Equation (2)):
  (2) $$F_{\mathrm{iden}}=F$$
2. Multi-scale cavity integral branch (Formula (3)):
  Perform parallel depth-separable convolutions in each channel of *F* with expansion rates of 1, 2, and 3. Denote the corresponding weights as $W_{\mathrm{dil}}^{\left(d\right)}$ (Equation (3)):
  (3) $$F_{\mathrm{dil}}^{\left(d\right)}\left[b,c\right]=W_{\mathrm{dil}}^{\left(d\right)}{*}_{d}F\left[b,c\right],\;d=1,2,3$$
  then Concatenate $\left\{F_{\mathrm{dil}}^{\left(1\right)},F_{\mathrm{dil}}^{\left(2\right)},F_{\mathrm{dil}}^{\left(3\right)}\right\}$ along the channel dimension to obtain $F_{\mathrm{dil}}\in R^{B\times C_{m}\times H\times W}$. Multi-scale dilated convolutions effectively expand the receptive field through a depthwise separable convolution structure, enhancing the model’s feature extraction ability while avoiding a significant increase in computational cost. The comparison of receptive fields is shown in Figure 3.
3. DoG Edge Extraction Branch:
  Apply two-scale Gaussian blurring $G(\cdot ;\sigma_{1})$ and $G(\cdot ;\sigma_{1})$ to *F* (Equation (4)).
  (4) $$F_{\mathrm{DoG}}=G\left(F;\sigma_{1}\right)-G\left(F;\sigma_{2}\right)\;$$
  Then, perform global average pooling on $F_{\mathrm{DoG}}$ and use a one-dimensional convolution with a kernel size of $k_{\mathrm{ECA}}$ and a sigmoid function to obtain the channel attention weights $\alpha \in R^{B\times C_{m}}$ (Equation (5)):
  (5) $$z=\mathrm{GAP}\left(F_{\mathrm{DoG}}\right),\;\alpha =\sigma \left(\mathrm{Conv}1D\left(z;k_{\mathrm{ECA}}\right)\right)\;$$
  Broadcast α along the spatial dimension, weighting $F_{\mathrm{DoG}}$ channel-wise. Then, map back to $C_{m}$ dimensions using a 1×1 convolution $W_{\mathrm{DoG}}$, yielding $F_{\mathrm{DoG}-\mathrm{out}}\in R^{B\times C_{m}\times H\times W}$. At this point, the three parallel outputs are as follows:
  $$F_{\mathrm{iden}},\;F_{\mathrm{dil}},\;F_{\mathrm{DoG}-\mathrm{out}}\in R^{B\times C_{m}\times H\times W}$$
  $F_{\mathrm{iden}}$ retains the original local information; $F_{\mathrm{dil}}$ extracts multi-scale spatial context; and $F_{\mathrm{DoG}-\mathrm{out}}$ enhances high-contrast edge features (Figure 4). Collectively, these three parallel outputs provide complementary semantic and detail information for subsequent concatenation and attention fusion.

### 3.3. Feature Fusion

Next, all features will be fused. The subsequent channel and spatial attention modules will then perform efficient adjustments to the weights of the fused features. This ensures that, regardless of whether edge information comes from a single or multiple scales, the model can perform higher-level optimization and focus on the final, combined features.

#### 3.3.1. Feature Concatenation

Concatenate the three feature streams above along the channel dimension. Then, use a 1×1 convolution to reduce the number of channels back to $C_{m}$ (Equation (6)):

(6) $$F_{cat}=Concat\left(F_{iden},F_{dil},F_{DoG_{o}ut}\right),\;F_{fuse_{i}nit}=SiLU\left(BN(W_{fuse}*F_{cat})\right)$$

where $W_{fuse}\in R^{C_{m}\times 3C_{m}\times 1\times 1}$.

#### 3.3.2. Channel Attention

Let $F_{ca\_in}=F_{fuse\_init}\in R^{B\times C_{m}\times H\times W}$ be the input feature tensor. First, perform both global average pooling and global max pooling along the spatial dimensions to obtain two channel descriptors $z_{avg},z_{max}\in R^{B\times C_{m}}$. These are then fed into a shared two-layer MLP: the first layer reduces the channel dimension from $C_{m}$ to $C_{m}/r$, and the second layer maps it back to $C_{m}$. A sigmoid activation is applied to the sum of the two MLP outputs, which yields the channel attention weights $\beta \in R^{B\times C_{m}}$ (Equation (7)):

(7) $$m_{avg}\;=\;\mathrm{MLP}\left(z_{avg}\right),\;m_{max}\;=\;\mathrm{MLP}\left(z_{max}\right),\;\beta \;=\;\sigma \left(m_{avg}+m_{max}\right)$$

Finally, broadcast β over the spatial dimensions and multiply it (channel-wise) with $F_{ca\_in}$, producing the refined output $F_{ca_{o}ut}\in R^{B\times C_{m}\times H\times W}$.

#### 3.3.3. Spatial Attention

Let $F_{sa\_in}=F_{ca\_out}\in R^{B\times C_{m}\times H\times W}$. First, apply average pooling and max pooling along the channel dimension to obtain two spatial maps $M_{avg},M_{max}\in R^{B\times 1\times H\times W}$. Concatenate these two maps along the channel axis, and then apply a 7×7 convolution followed by a sigmoid activation to obtain the spatial attention weights $\gamma \in R^{B\times 1\times H\times W}$(Equation (8)):

(8) $$\gamma \;=\;\sigma \left({\mathrm{Conv}}_{7\times 7}\left([\;M_{avg};\;M_{max}\;]\right)\right).$$

Broadcast γ over the channel dimension and multiply it (element-wise) with $F_{sa\_in}$, yielding the final fused feature map $F_{fused}\in R^{B\times C_{m}\times H\times W}$.

### 3.4. Output Mapping and Downsampling

Based on the fused feature map $F_{\mathrm{fused}}\in R^{B\times C_{m}\times H\times W}$, we apply a 3×3 convolution to map the channel dimension to $C_{\mathrm{out}}$. If downsampling is required, we set the stride s=2; otherwise, we keep the stride s=1 to preserve the spatial resolution (Equation (9)):

(9) $$Y=SiLU\left(BN(W_{out}{*}_{s}F_{fused})\right),\;W_{out}\in R^{C_{out}\times C_{m}\times 3\times 3}$$

where *s* denotes a convolution with stride *s*. When performing downsampling, s=2 and padding is set to 1; when not downsampling, s=1 and padding is also 1. This choice of padding guarantees that the output’s spatial dimensions become (H/s,W/s).

### 3.5. Module Visualization Results

**Figure 5:**
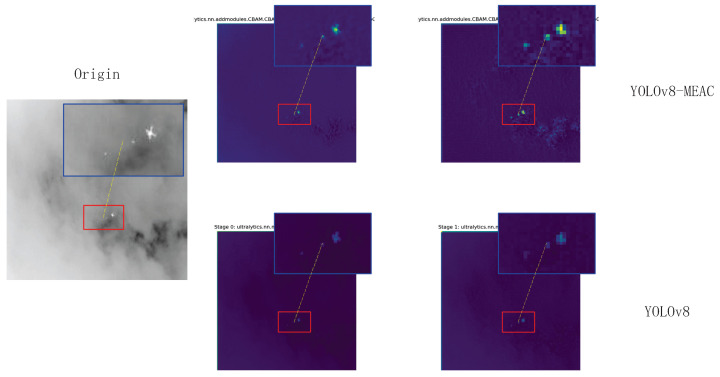
Feature maps.

**Figure 6:**
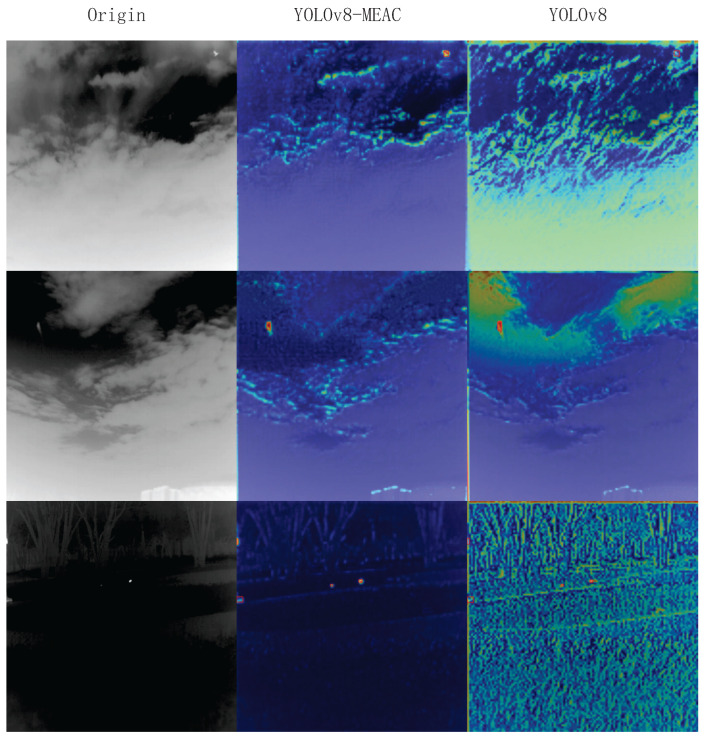
Heat maps.

To more intuitively observe MEAC’s ability to extract and retain features, feature maps (Figure 5) and heat maps (Figure 6) were randomly sampled for extraction.

As shown in the figure, the feature and heat maps generated by the MEAC module after processing indicate that the model’s object detection performance in complex environments has significantly improved compared to the original model. The specific advantages are reflected in the following aspects:

- **Enhanced feature discrimination capability:** MEAC generates clearer feature maps, which makes the separation between foreground objects (objects of interest) and the background more distinct. This is evident in the higher intensity and more concentrated spatial distribution of the ’hotspot’ regions of target objects.
- **Effective suppression of background noise:** MEAC can significantly suppress background noise and non-critical texture information, such as complex patterns in cloudy skies or dense foliage. This greatly reduces the risk of interference from non-target elements.
- **Enhanced environmental robustness:** MEAC demonstrates outstanding performance in highly challenging environments, such as those with complex backgrounds (e.g., high-intensity clouds) or low visibility (e.g., nighttime scenes). It effectively avoids the performance degradation issues caused by excessive background activation in standard models.
- **Optimized feature retention capability:** A comparison of the first feature map clearly shows that MEAC extracts key features more effectively and ensures they are well-preserved throughout the processing workflow. This lays the foundation for more robust object detection.

## 4. Experiment

### 4.1. Experimental Environment and Evaluation Criteria

The configuration of the experimental environment is shown in the Table 1.

The training parameters are as follows: the training cycle is 100 epochs; the batch size is 32; and the image size is 640×640. The model uses the stochastic gradient descent (SGD) optimizer for parameter optimization with an initial learning rate of 0.01 and a momentum parameter of 0.937. To prevent overfitting, the model uses a weight decay strategy with a weight decay value of $5\times 10^{-4}$. There are also some necessary settings for the experiments as shown in Table 2.

This paper’s experimental objective is to verify each convolutional module’s ability to extract target features in shallow layers. Therefore, the convolutional modules in the first two layers of the official P2 model of YOLOv8n were replaced. To evaluate the model’s effectiveness, this paper uses the evaluation metrics precision P, recall rate R, mAP50, and mAP50-95 (Equations (10)–(12)) and compares the model’s parameters and runtime speed using Params and GFLPOS.

(10) $$\mathrm{Precision}=\frac{\mathrm{TP}}{\mathrm{TP}+\mathrm{FP}}$$

(11) $$\mathrm{Recall}=\frac{\mathrm{TP}}{\mathrm{TP}+\mathrm{FN}}$$

(12) $$\mathrm{mAP}=\frac{1}{N}\sum_{i=1}^{N}{\mathrm{AP}}_{i}$$

TP (true positive) represents the number of correctly predicted true instances; FP (false positive) represents the number of incorrectly predicted true instances; and FN (false negative) represents the number of incorrectly predicted non-true instances. AP denotes average accuracy per category, and N denotes the total number of categories.

### 4.2. Datasets

**Figure 7:**
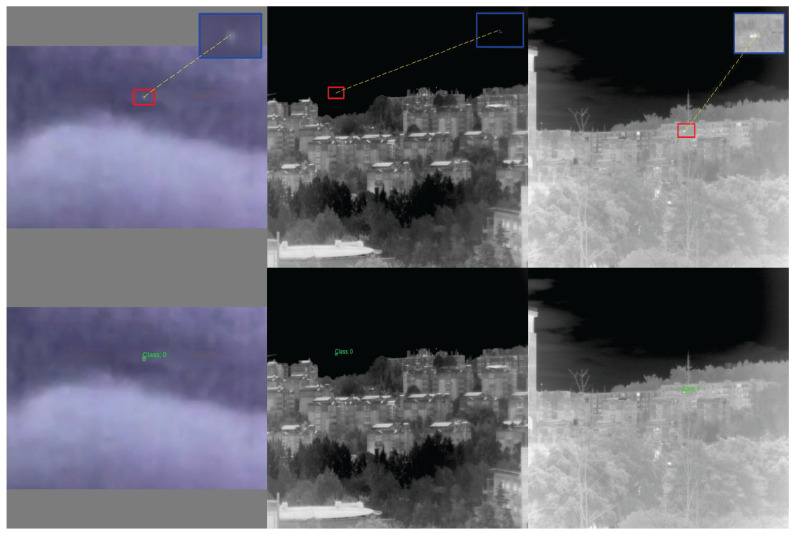
Dataset annotation.

This paper selected three commonly used public infrared small-target detection benchmark datasets: IRSTD-1K, SIRST-UAVB, and IRSTDv1, and an M3FD dataset for generalization testing. Figure 7 illustrates the annotation process for these datasets. The specific sources and characteristics of these datasets are as follows:

1. The IRSTD-1K [35] dataset was proposed by Zhang Mingjin et al. from Xi’an University of Electronic Science and Technology and contains 1001 infrared images with a resolution of 512 × 512. These images cover various target types, including drones, birds, ships, and vehicles. Due to the use of multispectral imaging, small targets appear extremely small and have blurred edges in environments with a low signal-to-noise ratio, creating a typical “multispectral imaging + small target” scenario with high background complexity and low contrast.
2. The SIRST-UAVB [43] dataset was proposed by Yang Jiangnan et al. from Southwest University of Science and Technology. This dataset includes 3000 images with a resolution of 640 × 512 and focuses primarily on small flying targets, such as drones and birds. The scene background is similarly complex with a low signal-to-noise ratio (SNR) and signal-to-clutter ratio (SCR). This makes small targets prone to being obscured by the background, which increases detection difficulty.
3. IRSTDv1 [44] was proposed by Dai Yimian et al. from Nanjing University of Aeronautics and Astronautics. This dataset includes 427 images of varying resolutions, with the highest resolution reaching 418 × 388. The targets are primarily small unmanned aerial vehicles. The main challenges of this dataset are the small target size, even at high resolutions, coupled with severe low-contrast background interference and similar textures between targets and backgrounds. Table 2 lists the basic information and main challenges of the aforementioned three datasets.
4. M3FD [45] was proposed by a team from Dalian University of Technology at the 2022 CVPR conference. The dataset contains 4200 images with a resolution of 1024 × 768. It presents challenges such as low contrast at high resolution, background interference, and target shapes that are similar to the background, aiming to address the robustness of object detection in complex environments (such as at night or in foggy conditions). The primary targets include common objects such as people, cars, buses, motorcycles, streetlights, and trucks.

The highly complex background interference and weak target features in each dataset effectively validate the detection performance and robustness of the proposed MEAC module for infrared small targets across various scenarios.

### 4.3. Ablation Experiments

To validate the effectiveness of each sub-structure in the MEAC module, this section conducts ablation experiments on the following branches under the same training settings: DoG edge (DoGEdge), Dilated (Dilated), Direct mapping (Identity). The experiments were conducted on three public datasets: IRSTD-1K, SIRST-UAVB, and IRSTDv1. The ablation schemes include

- Complete model (DoGEdge + Dilated + Identity).
- Remove DoGEdge (only retain Dilated and Identity); and 3.
- Remove the Dilated layer (only retain the DoGEdge and Identity layers).
- Remove Identity (only retain DoGEdge and Dilated).
- Baseline model (conventional convolution).

**Figure 8:**
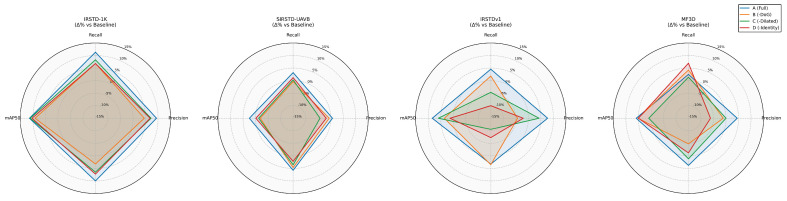
Radar plot.

Table 3, Table 4, Table 5 and Table 6 and Figure 8 summarize the quantitative results (precision, recall, mAP50, and mAP50-95) of each scheme on the IRSTD-1K, SIRST-UAVB, IRSTDv1 and MF3D datasets.

#### 4.3.1. Quantitative Ablation Results

1. Removal of DoG Edge Branches (Dilated + Identity Only)
  After removing the DoG edge branches, both precision and recall decreased significantly across the three datasets, particularly on IRSTD-1K. Precision dropped by 4.0%, and mAP50-95 decreased by 2.7%. This indicates that the DoG edge branches play a crucial role in amplifying high-frequency edge features. Without this branch, the model’s response to low-contrast or weakly textured small targets weakens. This leads to increased false negatives and false positives; on IRSTDv1, precision drops by 10.6%. This further demonstrates that DoGEdge is crucial for maintaining the contours of shallow targets. It significantly improves the localization accuracy and confidence of detection bounding boxes.
2. Removal of the Dilated Convolution Branch (DoG Edge + Identity Only)
  After removing the dilated branch, recall decreased significantly across all datasets. Recall decreased by 8.1%, and mAP50-95 decreased by 5.9% on IRSTDv1. This indicates that dilated convolutions provide a larger receptive field and fuse richer contextual information at shallow layers. This effectively improves the detection capability and localization accuracy of small targets in complex backgrounds. Without this branch, the network struggles to perceive both targets and their backgrounds simultaneously at shallow layers. This leads to missed detections and localization errors in scenarios with dense multiobjects or background false hotspots.
3. Removing the direct mapping branch (DoGEdge + Dilated only)
  Removing the identity branch significantly degrades model performance, particularly on IRSTDv1, where recall decreases by 12.8%, and precision decreases by 8.6%. The direct mapping branch provides a lossless channel between the main trunk and higher-level features. This enables the complete transmission of low-level texture details from the first layer to the second layer and beyond. When this branch is removed, small-target information in the shallow layers becomes overly compressed or smoothed. This causes small target responses to decay and edges to blur in the high-level feature maps. Consequently, detection accuracy and recall are significantly reduced.
4. Baseline Model Comparison
  The complete model significantly outperforms the baseline on all three datasets. mAP50 improves by 9.1% on IRSTD-1K and by 4.0% on IRSTDv1. DoGEdge contributes most to improving precision and localization accuracy, while Dilated is most effective in improving recall and suppressing false negatives. Identity ensures the propagation of original features from the shallow layer, which is indispensable for overall training stability and detection accuracy.
  The results of the ablation experiment show that adding each module positively impacts model performance, especially in terms of improving accuracy, recall, and mAP50-95. The DoGEEdge module is essential for enhancing edge details and detecting small objects, while the Dilated and Identity modules are critical for multi-scale feature extraction and maintaining model stability, respectively. Integrating all modules enables the model to demonstrate strong detection capabilities across various scenarios.
5. Generalization experiments on the dataset MF3D
  The results of the ablation experiment show that adding each module positively impacts model performance, especially in terms of improving accuracy, recall, and mAP50-95. The DoGE module is essential for enhancing edge details and detecting small objects, while the Dilated and Identity modules are critical for multi-scale feature extraction and maintaining model stability, respectively. Integrating all modules enables the model to demonstrate strong detection capabilities across various scenarios.
  Overall, combining all three modules significantly enhances the model’s performance in complex scenarios, making it the recommended configuration for practical applications.

#### 4.3.2. Qualitative Analysis

**Figure 9:**
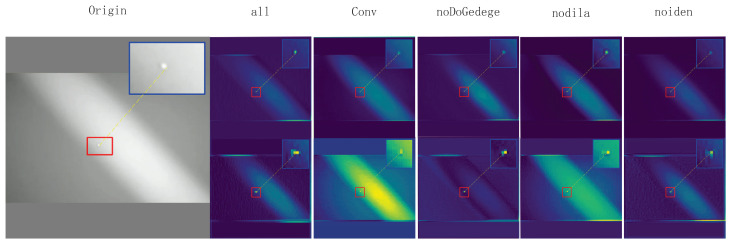
Feature map comparison.

To further reveal the role of each branch in the shallow feature extraction process, a feature map visualization comparison is used to show the feature maps of the first and second layers under different ablation configurations (see Figure 9).

1. The DoG edge branches amplify shallow, high-frequency information. After removing the DoG Edge branch and retaining only the Dilated + Identity branch, the edge signals in the second-layer feature maps at the same positions are significantly weakened (Figure 9, no edge column). This makes small targets prone to blending with the background and causes subsequent layers to more easily lose the target. In other experiments with the DoG Edge branch enabled, the contours of small targets in the second-layer feature maps are presented in high-contrast, bright colors (Figure 9), even when target contrast is extremely low. This allows clear edges to be retained in the shallow layers.
2. Multi-scale fusion is realized without downsampling by the hollow roll integral branch. After removing Dilated (while retaining DoG Edge + Identity), the activation of small targets in the second-layer feature map becomes loose and fragmented (Figure 9, noDilate), and the contrast between targets and their surroundings decreases. This indicates that the lack of cross-regional context leads to incomplete responses to weak, small targets in shallow layers. When Dilated is enabled, the second-layer feature map can simultaneously “see” small targets and the surrounding, larger background without reducing resolution. This forms concentrated, coherent activation regions.
3. Direct mapping branches ensure low-level detail propagation. After removing Identity (while retaining DoGEdge and Dilated), the target response in the second-layer feature map significantly decreases, and the edges become blurred (Figure 9, noiden). This leads to over-smoothing in subsequent layers, resulting in missed detections or inaccurate localization. When Identity is enabled, the low-level details extracted in the first layer can be directly transferred to the second layer. This enables small targets to retain geometric structure and contour information in higher layers.

#### 4.3.3. Summary

This section systematically verifies the necessity of the DoG edge branch, the hole convolution branch, and the direct mapping branch through rigorous ablation experiments and visual comparisons. The three branches jointly construct an “edge amplification + multi-scale context + original information flow” feature representation in the first two convolutional layers. This representation provides high-quality features for small objects for subsequent deep refinement and detection heads.

Quantitatively, removing any sub-branch results in a decline in precision, recall, and mean average precision (mAP). Qualitatively, removing any branch leads to significant degradation in the performance of small targets’ edges and activations in shallow feature maps. The synergistic interaction of the three components enables the complete model to achieve comprehensive improvements of 3–9% on the IRSTD-1K, SIRST-UAVB, and IRSTDv1 datasets. This validates the effectiveness of the proposed improvements in the field of infrared small target detection.

### 4.4. Comparison of MEAC Module Insertion in Different Models and Positions

To investigate MEAC’s performance in various architectures and positions further, we ran experiments with three state-of-the-art, official P2 models: YOLOv10, YOLOv11, and YOLOv12. We also used the head (place1), tail (place2), and neck (place3) networks of the backbone. The results are as follows (Table 7):

#### Conclusion Analysis

From the results of the three experimental groups, the sensitivity of different YOLO models to the insertion position of the MEAC module varies:

1. **YOLOv10n:** Inserting MEAC between the end of the backbone and the beginning of the neck (place2) yields the most balanced and significant improvements. In particular, mAP50 increases by approximately +6.77% on IRSTDv1 and +5.13% on IRSTD-1k, and by +2.80% on SIRST-UAVB. Recall and mAP50-95 also reach their highest values. When placed at the network front (place1), gains in Precision are the most consistent (approximately +3.37% and +4.18%), whereas placing MEAC at the rear of the neck (place3) yields slightly better improvements in mAP50 and Recall on the UAVB scenario.
2. **YOLOv11n:** The optimal insertion shifts to the network front (place1). On IRSTDv1, all four metrics achieve their maximum gains: Precision +4.85%, Recall +11.66%, mAP50 +4.45%, and mAP50-95 +4.54%. On SIRST-UAVB, performance improvements are even more pronounced (Precision +9.44%, Recall +10.11%, mAP50 +9.67%, mAP50-95 +5.79%). Although place2 yields the highest mAP50 gain (+3.31%) on IRSTD-1k, the overall performance remains inferior to place1.
3. **YOLOv12n:** Likewise, inserting MEAC at the network front (place1) provides the most significant absolute gains. On IRSTDv1: Precision +1.24%, Recall +1.58%, mAP50 +5.92%, mAP50-95 +6.71%. On IRSTD-1k: Precision +1.13%, Recall +3.55%, mAP50 +3.78%, mAP50-95 +5.15%. On SIRST-UAVB: Precision +3.06%, Recall +3.10%, mAP50 +2.89%, mAP50-95 +1.98%. In contrast, the gains at place3 are weakest, and place2 occasionally even shows slight negative improvements in some scenarios.

Overall, although different YOLO model scales exhibit distinct preferences for MEAC insertion positions, some general patterns emerge. For YOLOv10n, inserting MEAC at place2 maximizes overall mAP50 and mAP50-95. For larger models YOLOv11n and YOLOv12n, placing MEAC at the network front (place1) delivers the most pronounced gains in Precision, Recall, mAP50, and mAP50-95. If a balance between recall and precision is desired, the rear of the neck (place3) is a viable alternative. Importantly, regardless of insertion position, our MEAC module consistently improves metrics with only a slight increase in computational cost and parameter count.

### 4.5. Comparison with Common Convolution Modules

To validate the effectiveness of MEAC on different convolution operators, this section conducts a comparative analysis of 11 mainstream convolution modules. These operators include ACmix [39], AKConv [46], DRConv [47], DSConv, LSKConv [48], MixConv [49], PConv [43], standard convolution (Conv), ODConv [38], GConv [41], and Involution [17]. Experiments were conducted on three datasets, IRSTD-1K, SIRST-UAVB, and IRSTDv1, using the evaluation metrics Precision (P), Recall (R), mAP50, mAP50-95, Params (M), and Gflops/G. The results are as follows (Table 8, Table 9 and Table 10).

#### 4.5.1. Quantitative Analysis of Comparative Experiments

1. Overall Performance Comparison. On three datasets, MEAC outperforms other operators in all key metrics (P, R, and mAP50 and mAP50-95). MEAC demonstrates a particularly significant advantage in mAP50-95 (high IoU threshold), indicating its superior performance in precisely localizing small edge targets. 2. Analysis of MEAC’s Advantages.

- Edge Perception and Multi-Scale Fusion:
  MEAC combines DoG edge responses with multi-scale dilated convolutions to extract target contours in low-contrast and low-SNR environments.
- Lightweight Design:
  With 2.92 million parameters and a computational load of only 6.8 GFlops, MEAC is highly efficient. It maintains a lightweight structure while achieving an FPS of 191.6, which is significantly higher than that of similar operators, such as ACmix (87.8 FPS) and Involution (110.1 FPS). MEAC also maintains good inference speed among mainstream operators, demonstrating good real-time performance and deployment friendliness.
- High-Precision Localization:
  At high IoU thresholds, MEAC’s mAP50-95 significantly outperforms other operators, indicating its heightened sensitivity to the boundaries of small infrared targets.

#### 4.5.2. Qualitative Analysis of Comparative Experiments

**Figure 10:**
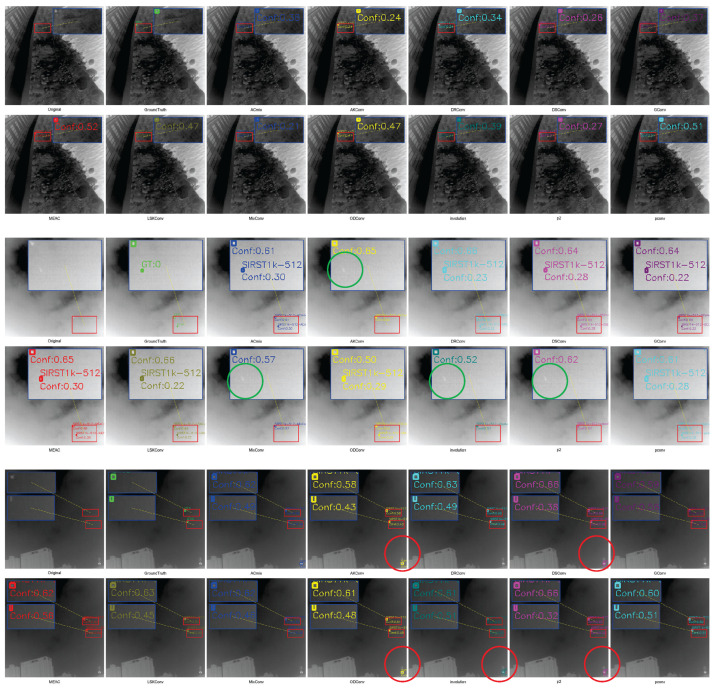
IRSTD-1K sample test results.

**Figure 11:**
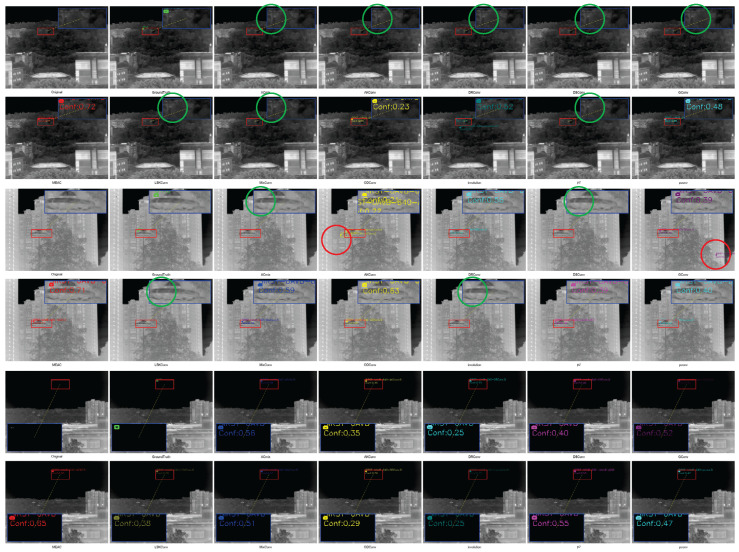
SIRST-UAVB sample test results.

**Figure 12:**
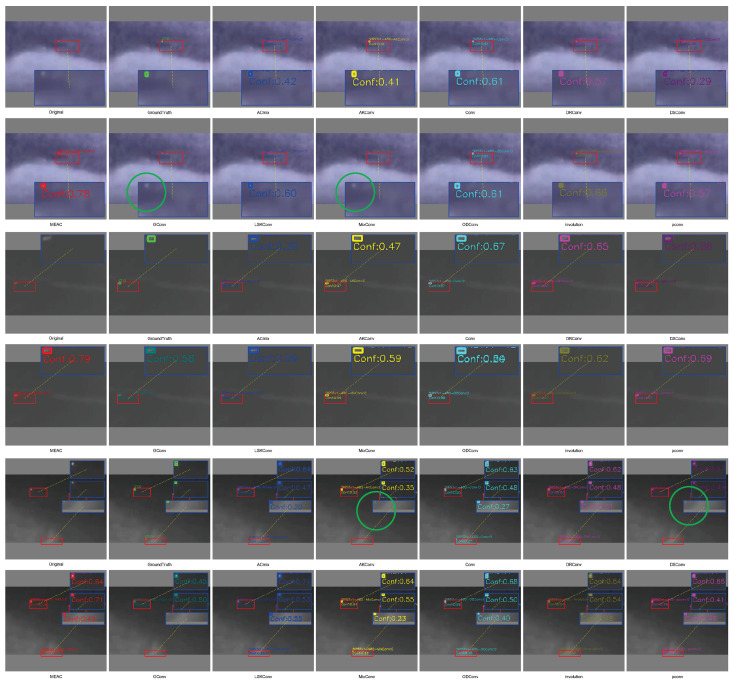
IRSTDv1 sample test results.

We compared the detection results of each convolutional module by selecting representative samples from each dataset for visualization (Figure 10, Figure 11 and Figure 12), where green circles indicate missed detections and red circles indicate false detections.

1. IRST-UAVB Dataset. MEAC achieves nearly perfect detection of extremely small targets, even when target sizes are only a few pixels. Their contours are amplified and correctly localized in shallow features. Other operators are prone to false negatives or misclassifying background as targets amidst complex background artifacts.
2. IRSTD-1K Dataset. Due to the low signal-to-noise ratio of multi-spectral imaging, conventional operators often overlook weak signals. However, MEAC accurately detects most targets with high confidence through the synergistic effects of the DoG branch, which enhances edges, and the Dilated branch, which captures multi-scale context.
3. IRSTDv1 Dataset. The target-to-background contrast is extremely low, and edge blurring is severe. Other operators often misclassify weak signals as noise and fail to detect them in this scenario. MEAC uses a multi-scale edge perception mechanism to accurately capture target contours while maintaining a low false detection rate. This demonstrates its robustness and generalization capability in extreme scenarios.

This section compares the performance of the MEAC method with that of 11 other mainstream convolutional modules on three small target infrared datasets. The results demonstrate that MEAC outperforms other methods in all metrics, particularly in its high-precision detection of small targets at high IoU thresholds. These results fully validate the effectiveness and practical value of MEAC in the field of infrared small target detection through its edge-aware, multi-scale fusion, and dual attention mechanisms.

## 5. Conclusions

The multi-scale, edge-aware convolution (MEAC) module proposed in this paper effectively enhances the ability to represent features of small infrared targets by integrating weak target details, multi-scale context, and high-contrast edge information through parallel fusion and by combining channel and spatial attention mechanisms.

Ablation experiments demonstrate that the model achieves significantly better detection performance on major public datasets when the DoG edge branch, hollow convolution branch, and direct mapping branch are fully integrated compared to the baseline using conventional convolutions alone. Removing any single branch results in a noticeable decline in relevant metrics, proving the three branches’ complementary nature and necessity in transmitting weak small target details, extracting multi-scale information, and enhancing edge responses.

Experiments comparing MEAC with various mainstream convolution operators (e.g., separable convolution, low-rank convolution, hybrid convolution, and learnable edge extraction operators) further validate MEAC’s advantages. On datasets with different levels of difficulty and background complexities, networks integrated with MEAC consistently outperform other methods in key metrics such as accuracy, recall rate, and overall mean average precision (mAP). MEAC has the same number of parameters as conventional convolutions and only slightly increases computation. However, it still has a significant advantage over computationally intensive operators. This indicates that the MEAC module achieves stronger perception of small infrared targets and background interference suppression while ensuring lightweight, high-efficiency performance.

Thanks to its flexible structural design and efficient performance, MEAC is suitable not only for infrared small target detection tasks, but also for other scenarios where computational resources are limited but high detection accuracy is required. These scenarios include medical image analysis, autonomous driving, security surveillance, and aerial remote sensing. By replacing traditional convolutional operators in these applications, MEAC balances detection accuracy and computational overhead effectively. With the introduction of further optimization techniques, such as model compression and quantization, MEAC is expected to evolve into a key module for various embedded systems and real-time applications in the future. This will facilitate the practical deployment and application of related technologies.

In summary, the MEAC module significantly improves the performance and robustness of infrared small-target detection by synergistically fusing multi-source information and calibrating features via attention. This demonstrates its excellent generalization capabilities and serves as an efficient alternative to conventional modules. Future work may explore the following directions:

- Using pruning, quantization, or depth separability techniques reduces model size and computational overhead, meeting the requirements of embedded or real-time systems. These optimizations allow MEAC to maintain high performance while adapting to resource constraints in practical applications.
- Integrate the MEAC module with long-range dependency modeling architectures, such as Transformers, to enhance cross-scale information interaction and global context understanding.
- Extend the application of the module to different small-target detection domains, such as medical imaging and aerial remote sensing, to validate its generalizability and practical benefits. Through these improvements and extensions, MEAC is expected to enhance small-target detection performance in complex environments and advance the practical application of related technologies.

## Figures and Tables

**Table 1:** Experimental environment configuration.

**Operating System**	Ubuntu 22.04
**GPU**	RTX 4090 (24 GB)
**CPU**	16 VCPU Intel(R) Xeon(R) Platinum 8352V CPU @ 2.10 GHz
**Memory**	120 GB
**Programming Languages**	Python 3.10
**Frameworks**	PyTorch 2.1.0 + CUDA 12.1
**IDE**	JupyterLAb

**Table 2:** Experimental setup.

Training/Validation/Test Split	Random Seed
80%/10%/10%	seed: 0
**Data Augmentation**	**Initialization**
YOLO official Closmic settings (set to 10)	Random Initialization

**Table 3:** Detection performance on IRSTD-1K.

DoGE	Dilated	Identity	P	R	mAP50	mAP50-95
✓	✓	✓	**89.8%**	**83.4%**	**89.3%**	**44.0%**
	✓	✓	85.8%	80.0%	87.5%	41.3%
✓		✓	88.0%	81.1%	89.1%	42.6%
✓	✓		87.7%	80.0%	88.5%	42.9%
-	-	-	82.1%	74.9%	80.2%	40.0%

**Table 4:** Detection performance on SIRSTD-UAVB.

DoGE	Dilated	Identity	P	R	mAP50	mAP50-95
✓	✓	✓	**85.5%**	**77.6%**	**82.9%**	**38.5%**
	✓	✓	84.4%	74.9%	79.4%	38.1%
✓		✓	81.3%	75.4%	79.9%	37.7%
✓	✓		83.4%	76.1%	80.8%	37.2%
-	-	-	84.9%	75.2%	80.9%	36.4%

**Table 5:** Detection performance on IRSTDv1.

DoGE	Dilated	Identity	P	R	mAP50	mAP50-95
✓	✓	✓	**95.2%**	**91.8%**	**94.7%**	**44.0%**
	✓	✓	84.6%	89.4%	90.5%	**44.1%**
✓		✓	92.2%	83.7%	92.6%	38.1%
✓	✓		86.6%	79.0%	88.5%	39.5%
-	-	-	88.4%	87.8%	87.4%	42.6%

**Table 6:** Detection performance on MF3D.

DoGE	Dilated	Identity	P	R	mAP50	mAP50-95
✓	✓	✓	**84.1%**	**64.7%**	**74.2%**	**52.1%**
✓	✓	-	75.5%	67.5%	73.6%	49.6%
✓	-	✓	80.5%	64.0%	70.7%	50.8%
-	✓	✓	79.6%	65.8%	73.7%	47.8%
-	-	-	80.5%	63.13%	70.1%	50.2%

**Table 7:** Comprehensive performance comparison across models and MEAC positions.

IRSTDv1							
Model	Configuration	Precision	Recall	mAP50	mAP50-95	GFLOPs	Params (M)
YOLOv10n	Baseline	79.5%	76.8%	78.9%	32.2%	7.8	2.83
	MEAC_place1	82.9%	72.6%	82.2%	36.9%	8.5	2.83
	MEAC_place2	73.0%	85.9%	85.7%	37.2%	8.6	2.82
	MEAC_place3	80.8%	79.6%	84.0%	34.5%	8.3	2.84
YOLOv11n	Baseline	83.8%	76.8%	84.5%	36.0%	5.2	2.66
	MEAC_place1	88.7%	88.5%	89.0%	40.5%	5.8	2.67
	MEAC_place2	87.9%	82.2%	88.5%	37.4%	5.5	2.77
	MEAC_place3	85.8%	85.5%	88.8%	38.3%	5.3	2.62
YOLOv12n	Baseline	87.3%	83.3%	83.9%	34.5%	6.4	2.73
	MEAC_place1	88.5%	84.9%	89.8%	41.2%	7.1	2.73
	MEAC_place2	89.0%	84.0%	84.3%	37.3%	6.7	2.83
	MEAC_place3	81.4%	90.5%	86.1%	37.2%	6.6	2.68
**IRSTD-1K**							
YOLOv10n	Baseline	78.6%	78.6%	81.2%	37.9%	7.8	2.83
	MEAC_place1	82.8%	76.4%	86.2%	41.6%	8.5	2.83
	MEAC_place2	85.2%	77.9%	86.4%	42.5%	8.6	2.82
	MEAC_place3	81.3%	77.9%	83.6%	39.0%	8.3	2.84
YOLOv11n	Baseline	87.5%	83.5%	87.4%	40.9%	5.2	2.66
	MEAC_place1	88.6%	82.1%	89.3%	42.6%	5.8	2.67
	MEAC_place2	87.3%	85.6%	90.7%	41.1%	5.5	2.77
	MEAC_place3	83.4%	85.5%	88.4%	41.8%	5.3	2.62
YOLOv12n	Baseline	86.1%	81.0%	84.1%	38.6%	6.4	2.73
	MEAC_place1	87.2%	84.6%	87.9%	43.8%	7.1	2.73
	MEAC_place2	83.9%	82.9%	86.9%	41.9%	6.7	2.83
	MEAC_place3	85.8%	81.4%	87.1%	40.7%	6.6	2.68
**SIRST-UAVB**							
YOLOv10n	Baseline	70.9%	67.6%	71.8%	33.0%	7.8	2.83
	MEAC_place1	71.8%	66.0%	73.3%	33.6%	8.5	2.83
	MEAC_place2	72.8%	68.0%	74.6%	34.0%	8.6	2.82
	MEAC_place3	76.9%	67.4%	75.1%	36.3%	8.3	2.84
YOLOv11n	Baseline	76.7%	62.8%	69.6%	29.9%	5.2	2.66
	MEAC_place1	86.1%	72.9%	79.3%	35.7%	5.8	2.67
	MEAC_place2	80.3%	69.0%	74.4%	33.3%	5.5	2.77
	MEAC_place3	80.0%	65.8%	73.6%	33.3%	5.3	2.62
YOLOv12n	Baseline	75.3%	62.9%	68.3%	31.1%	6.4	2.73
	MEAC_place1	78.4%	66.0%	71.2%	33.1%	7.1	2.73
	MEAC_place2	75.1%	65.6%	69.8%	32.1%	6.7	2.83
	MEAC_place3	67.4%	61.7%	66.1%	29.9%	6.6	2.68

**Table 8:** Comparison of convolution modules on IRSTD-1K.

Module	Params(M)	Gflops/G	FPS	P	R	mAP50	mAP50-95
ACmix	2.92	6.7	99.8	88.1%	80.0%	87.6%	39.6%
AKConv	3.60	24.2	213.6	81.2%	83.7%	85.9%	39.9%
DRConv	3.11	6.7	274.9	87.1%	82.1%	87.6%	42.1%
DSConv	2.92	**6.0**	**308.5**	**92.8%**	77.9%	87.9%	39.9%
LSKConv	3.10	6.5	243.4	84.2%	84.7%	85.3%	40.9%
MixConv	2.93	6.3	239.4	85.2%	79.6%	86.0%	40.2%
PConv	3.09	6.3	205.8	86.4%	**84.8%**	86.8%	41.7%
Conv	2.92	6.2	290.2	82.1%	74.9%	80.2%	40.6%
ODConv	2.96	6.1	204.1	86.7%	80.7%	84.8%	39.9%
GConv	2.92	6.2	259.9	85.1%	**86.4%**	87.7%	43.5%
Involution	2.94	6.2	217.8	89.3%	71.7%	86.1%	38.4%
Ours (MEAC)	**2.92**	**6.8**	**200.4**	**89.8%**	**83.4%**	**89.3%**	**44.0%**

**Table 9:** Comparison of convolution modules on SIRSTD-UAVB.

Module	Params(M)	Gflops/G	FPS	P	R	mAP50	mAP50-95
ACmix	2.92	6.7	104.0	84.1%	72.7%	78.7%	36.3%
AKConv	3.60	24.2	194.5	84.0%	70.3%	76.4%	33.9%
DRConv	3.11	6.7	294.1	77.5%	**77.8%**	78.4%	35.4%
DSConv	2.92	**6.0**	**337.6**	77.8%	72.3%	74.9%	33.1%
LSKConv	3.10	6.5	260.7	81.3%	76.4%	79.5%	35.7%
MixConv	2.93	6.3	311.0	83.0%	75.4%	79.9%	36.3%
PConv	3.09	6.3	274.4	83.2%	73.8%	78.5%	35.5%
Conv	2.92	6.2	331.8	80.4%	75.7%	80.5%	36.7%
ODConv	2.96	6.1	246.8	82.4%	75.2%	80.5%	36.3%
GConv	2.92	6.2	332.1	84.6%	76.0%	80.5%	36.5%
Involution	2.94	6.2	265.4	84.6%	63.2%	68.2%	29.7%
Ours (MEAC)	**2.92**	**6.8**	**221.8**	**85.5%**	**77.6%**	**82.9%**	**38.5%**

**Table 10:** Comparison of convolution modules on IRSTDv1.

Module	Params(M)	Gflops/G	FPS	P	R	mAP50	mAP50-95
ACmix	2.92	6.7	87.8	76.6%	75.9%	77.7%	29.5%
AKConv	3.60	24.2	224.4	85.4%	75.9%	83.5%	27.4%
DRConv	3.11	6.7	253.8	80.7%	87.0%	86.3%	31.8%
DSConv	2.92	**6.0**	222.0	79.9%	75.9%	76.9%	34.0%
LSKConv	3.10	6.5	193.7	81.3%	76.4%	79.5%	35.7%
MixConv	2.93	6.3	249.3	81.5%	81.5%	84.9%	30.8%
PConv	3.09	6.3	242.3	**92.1%**	85.8%	**89.6%**	33.4%
Conv	2.92	6.2	261.0	79.5%	83.7%	86.6%	**42.6%**
ODConv	2.96	6.1	195.9	86.5%	82.8%	84.3%	35.1%
GConv	2.92	6.2	**275.7**	83.6%	75.5%	80.0%	33.0%
Involution	2.94	6.2	110.1	84.8%	77.8%	81.5%	32.5%
Ours (MEAC)	**2.92**	**6.8**	**191.6**	**95.2%**	**91.8%**	**94.7%**	**44.0%**

## Data Availability

Data is contained within the article.
